# Osteochondral tissue engineering using a biphasic collagen/GAG scaffold containing rhFGF18 or BMP-7 in an ovine model

**DOI:** 10.1186/s40634-014-0013-x

**Published:** 2014-09-26

**Authors:** Alan Getgood, Frances Henson, Carrie Skelton, Roger Brooks, Hans Guehring, Lisa A Fortier, Neil Rushton

**Affiliations:** The Fowler Kennedy Sport Medicine Clinic 3M Centre, The University of Western Ontario, London, Ontario N6A 3K7 Canada; Department of Veterinary Medicine, University of Cambridge, Cambridge, UK; The University of Cambridge Orthopaedic Research Unit, Cambridge, UK; Merck Serono GmbH, Darmstadt, Germany; Department of Clinical Sciences, Cornell University, Ithaca, USA

**Keywords:** Osteochondral repair, Sheep, Biphasic scaffold, rhFGF18

## Abstract

**Background:**

The aim of this study was to investigate the effect of combining rhFGF18 or BMP-7 with a biphasic collagen/GAG osteochondral scaffold (Chondromimetic) on the repair of osteochondral defects in sheep.

**Methods:**

Osteochondral defects (5.8x6mm) were created in the medial femoral condyle (MFC) and the lateral trochlea sulcus (LTS) of the stifle joint of 24 female sheep. Sheep were randomly assigned to four groups (n = 6); 1) empty defect, 2) scaffold only, 3) scaffold + rhFGF-18 (30 μg) and 4) scaffold + BMP-7 (100 μg). At 6 months the defects underwent non-destructive mechanical testing, gross assessment of repair tissue (ICRS score) and histological analysis (Modified O’Driscoll score).

**Results:**

***ICRS repair score:*** Defects treated with scaffold + rhFGF18 (mean 9.83, 95% CI 8.43-11.23) and scaffold + BMP-7 (10, 9.06-10.94) in the MFC had significantly improved ICRS scores compared to empty defects (4.2, 0–8.80) (p = 0.002). ***Mechanical properties:*** BMP-7 treated defects (mean 64.35, 95% CI 56.88-71.82) were significantly less stiff than both the rhFGF18 (mean 84.1, 95% CI 76.8-91.4) and empty defects in the LTS, compared to both contralateral limb (p = 0.003), and the perilesional articular cartilage (p < 0.001). ***Histology:*** A statistically significant improvement in the modified O’Driscoll score was observed in the rhFGF18 treated group (mean 16.83, 95% CI 13.65-20.61) compared to the empty defects (mean 9, 95% CI 4.88-13.12) (p = 0.039) in the MFC. Excellent tissue fill, lateral integration and proteoglycan staining was observed. Only the rhFGF18 defects showed pericellular type VI collagen staining with positive type II collagen and reduced positive type I collagen staining. The majority of defects in the control and BMP-7 groups demonstrated fibrocartilagenous repair tissue.

**Conclusion:**

Statistically significant improvements in gross repair, mechanical properties and histological score were found over empty defects when Chondromimetic was combined with rhFGF18. These results suggest that rhFGF18 may play a significant role in articular cartilage repair applications.

## Background

There have been many technological advances in articular cartilage repair techniques over the past decade but the ability to regenerate hyaline cartilage continues to elude us. Procedures such as autologous chondrocyte transplantation and microfracture have, at best, been able to produce a repair tissue with hyaline like features [1]. More recently, a greater focus has been placed on the influence of subchondral bone in the disease process, with articular cartilage now being properly recognised as a composite structure [2]. As a result, tissue engineering principals are now starting to be applied to regenerate the osteochondral unit.

It has been demonstrated that osteochondral defects show improved repair parameters when a scaffold is used within the lesion [3]. Of the scaffolds used, a novel biphasic osteochondral scaffold (Chondromimetic, Orthomimetics/TiGenix, Cambridge, UK) has shown promise in early studies [4]. This collagen/chondroitin sulphate/calcium phosphate scaffold was demonstrated to induce healing with a repair tissue with features of hyaline cartilage at a 6 month end point in the caprine knee [4]. Whilst these results are encouraging, it is likely that a combination of a scaffold and bioactive molecules, such as chondrogenic growth factors, will enhance the healing process [3].

A number of growth factors have been tested both *in-vitro* and *in-vivo* for their chondrogenic properties, including insulin like growth factor 1 (IGF-1) [5] and bone morphogenetic protein 7 (BMP-7) [6,7]. More recently, fibroblast growth factor 18 (FGF18) has also been reported to promote chondrogenesis [8,9]. FGF-18 is a member of the FGF family, in which there are 23 members that bind to 4 structurally related high affinity FGF receptor (FGFR1-4) tyrosine kinases. The essential role of FGF’s in signalling in bone and cartilage metabolism is evident from genetic diseases, such as achondroplasia, which are associated with mutations in the FGFR gene.

Studies have shown that FGF18 acts as a ligand for the FGFR3 receptor, expressed on proliferating and prehypertrophic chondrocytes. Liu et al. [10] demonstrated that, in FGF18 knockout mice, the growth plate phenotype was similar to that of mice lacking FGFR3 – there was a delay in ossification and a reduced expression of osteogenic markers. They also showed that in FGF18/FGFR3 knockout mice, expanded zones of proliferating and hypertrophic chondrocytes were formed, with increased chondrocyte proliferation and differentiation. These negative effects in the growth plate are in contrast to the positive effect on mature chondrocytes. Ellsworth et al. have shown a significant direct effect of FGF18 on the growth and proteoglycan synthesis of mature murine and human chondrocytes [11]. An in-vivo study by Power et al. [12] demonstrated that the addition of recombinant human FGF18 (rhFGF18) to microfracture defects in sheep resulted in improved fill and quality of repair tissue with characteristics of hyaline cartilage.

It is therefore possible that FGF18 may play a role in the control of both chondrocyte and bone cell behaviour in tissue repair and warrants an investigation for its use in osteochondral regeneration.

This study was designed to investigate the effect of adding either rhFGF18 (Merck Serono, Darmstadt, Germany) or BMP-7 to a biphasic collagen-glycosaminoglycan (collagen-GAG) scaffold in an ovine osteochondral defect model. The null hypothesis was that neither the addition of rhFGF18 nor BMP-7 would improve the quality of osteochondral repair tissue when compared to a defect filled with scaffold alone.

## Methods

This study received approval from both local research ethics committee (University of Cambridge Biomedical Support Services) and the Home Office (United Kingdom Home Office).

*Animals:* A total of 24 skeletally mature female Welsh Mountain sheep between the ages of 3 and 5 years old were included in the study. Each treatment group contained six sheep (n = 6).

*Experimental design:* For all animals, full thickness osteochondral defects 5.8 mm wide by 6 mm deep were created in the proximal lateral trochlea sulcus (LTS) and in the medial femoral condyle (MFC) of the right stifle joint using custom made instrumentation. Four treatment groups were used; A - no scaffold in defect (‘empty defect’), B - scaffold alone (‘scaffold’), C - scaffold plus rhFGF18 (Merck Serono, Darmstadt, Germany, (‘scaffold + rhFGF18’) and D - scaffold plus BMP-7 (R&D systems, Abingdon, UK) (‘scaffold + BMP-7’).

*Animal anaesthesia, preparation and surgical technique:* Prior to surgery animals were selected at random and identification ear tags applied. All animals had food and water removed 24 hours before surgery. General anaesthesia was induced with an injection of thiopentone (3 mg/kg) into the external jugular vein. Maintenance was achieved via inhalational anaesthetic of a mixture of isofluorane, nitrous oxide and oxygen. Perioperative analgesia was provided by pre-operative intramuscular Carprofen (1.5 mg/ml) and antibiotic prophylaxis was given via intramuscular procaine penicillin (10 mg/ml).

The basic surgical procedure was identical for all subjects and performed under strict asepsis by a single surgeon. Each stifle was physically examined for any abnormalities whilst anaesthetised. No gross instability or pathology was found, therefore no animal was excluded from participation within the study.

The animal was placed in a dorsal recumbency position and, following surgical preparation, the right stifle joint opened via a lateral parapatellar approach. Following patella subluxation, the LTS defect was made 10 mm distal to the top of the lateral trochlear ridge aligned with the middle of the lateral trochlear groove. The MFC defect was made 10 mm distal to the condyle groove junction and aligned with the medial crest of the trochlear groove. A 5.8 mm wide × 6 mm deep cylindrical defect was created and a 6mm×6mm collagen-GAG scaffold plus appropriate test substance placed into the defect.

Test substances were reconstituted and added to the scaffold prior to commencing the skin incision. This allowed at least 10 minutes for the growth factor to adsorb on to the scaffold, prior to insertion into the defect. rhFGF-8 was reconstituted via the directions of the supplier with 0.9% saline and added to the scaffolds in a concentration of 150 μg/ml, providing 30 μg per scaffold. Lyophilised BMP-7 was reconstituted with 4 millimolar HCL and applied to the scaffold in a concentration of 500 μg/ml, providing 100 μg per scaffold. This concentration was chosen due to previous studies and following personal communication [6].

The joint was then cycled through a range of motion to ensure a satisfactory rim fixation of the plug, following which the joint was closed in a standard fashion. Postoperatively, animals were allowed to fully weight bear, but kept in small pens for 48 hours to reduce ambulation. All animals were housed indoors for the remaining study period in large pens, which allowed a moderate degree of ambulation. Regular checks were made for any animal displaying signs of postoperative discomfort with additional postoperative analgesia given if required. All further treatments were recorded as appropriate.

*Necropsy:* Animals were humanely sacrificed at 26 weeks postoperatively using a lethal dose of sodium pentobarbital.

Outcome measures:

1. **Gross Morphology** - The joints were opened and the surface of the osteochondral defect sites blindly scored by 2 observers using the International Cartilage Repair Society score (Table 1). The presence of degenerative change was noted as per the Outerbridge score (Table 2).
2. Mechanical testing - Each implant site underwent non-destructive mechanical testing to determine changes to the cartilage surface surrounding the implant or empty defect. Measurements were taken in duplicate from the centre of the osteochondral defect, and at a distance of 1 mm from the original edge of the created osteochondral defect at the 12, 3, 6, and 9 o’clock positions, using a handheld digital durometer (Shore S1, M scale, Instron Ltd, UK). A number between 0–100 (arbitrary unit) would be given (with an inbuilt calibrated error of +/−5) at each site and a mean calculated. These measurements were then repeated in the contralateral limb in the same anatomic sites. The stiffness of the reparative tissue was then expressed as percentage stiffness relative to the control cartilage in the contralateral limb. This therefore allowed a surrogate measure of stiffness to be made, as previously published [4].
3. **Histology** - Specimens were decalcified in formic acid/sodium citrate over two weeks and then processed for routine paraffin embedding. Sections of 7 μm thickness were made through the central portion of the defect. Sections were stained with Safranin O/Fast Green. One slide from the centre of each defect was blindly scored by two investigators using a modified O’Driscoll score (Table 3) [13].
4. **Immunohistochemistry** - Immunohistochemistry was performed as described previously [14]. The following primary antibodies were used in this study; monoclonal mouse anti human type I collagen (MP Biomedicals, US, 1 in 200 dilution), monoclonal mouse anti human type II collagen (MP Biomedicals, US, 1 in 100 dilution) and monoclonal mouse anti-rabbit type VI collagen (Abcam, UK, 1 in 500 dilution). Horseradish peroxidase-conjugated secondary anti-rabbit and mouse immunoglobulin were used as appropriate, and the colour reaction developed with 0.1% 3′, 3-diaminobenzidine tetrachloride (DAB)/0.01% hydrogen peroxide. Normal species-specific serum was used as a control in all experiments. The degree of positive staining for types I, II and VI collagens was evaluated by semi quantitative scoring on a scale of 1 to 4 for intensity i.e. inconspicuous (1), mild (2), moderate (3), and strong (4) [15]. In addition the location of type VI immunoreactivity was noted i.e. pericellular or territorial.

**Table 1:** **ICRS repair score** [16]

**Characteristic**	**Grading**	**Score**
**Degree of defect repair**	Level with surrounding cartilage	4
	75% repair of defect depth	3
	50% repair of defect depth	2
	25% repair of defect depth	1
	0% repair of defect depth	0
**Integration to border zone**	Complete integration with border zone	4
	Demarcating border <1 mm	3
	¾ of graft integrated, ¼ with notable border >1 mm	2
	½ of graft integrated with surrounding cartilage, ½ with a notable border >1 mm	1
	From no contact to 1/4th of graft integrated with surrounding cartilage	0
**Macroscopic appearance**	Intact smooth surface	4
	Fibrillated surface	3
	Small, scattered fissures or cracks	2
	Several, small or few but large fissures	1
	Total degeneration of grafted area	0
	**Total**	**12**

**Table 2:** **Outerbridge score for macroscopic degenerative change** [17]

**Outerbridge score**	**Explanation**
**Grade 0**	Normal
**Grade I**	Mild softening of the articular cartilage
**Grade II**	Fibrillation and fissuring of chondral surface
**Grade III**	Cracks in articular cartilage, no subchondral bone exposed
**Grade IV**	Full thickness chondral defects down to bone

**Table 3:** **Modified O’Driscoll Histology Score** [13]

	**Characteristic**		**Grading**	**Score**
**I**	**% Hyaline Cartilage:**		80-100	8
			60-80	6
			40-60	4
			20-40	2
			0-20	0
**II**	**Structural**	**A. Surface irregularity:**	Smooth and intact	2
	**Characteristics:**		Fissures	1
			Severe disruption, fibrillation	0
		**B. Structural integrity:**	Normal	2
			Slight disruption, including cysts	1
			Severe lack of integration	0
		**C. Thickness:**	100% of normal adjacent cartilage	2
			50-100% or thicker than normal	1
			0-50%	0
		**D. Bonding to adjacent cartilage:**	Bonded at both ends of graft	2
			Bonded at one end/partially both ends	1
			Not bonded	0
**III**	**Freedom from cellular changes of degeneration:**		Normal cellularity, no clusters	2
			Slight hypocellularity, <25% chondrocyte clusters	1
			Moderate hypocellularity, >25% clusters	0
**IV**	**Freedom from degenerate changes in adjacent cartilage:**		Normal cellularity, no clusters, normal staining	3
			Normal cellularity, mild clusters, moderate staining	2
			Mild or mod hypocellularity, slight staining	1
			Severe hypocellularity, slight staining	0
**V**	**Reconstitution of subchondral bone:**		complete reconstitution	2
			greater than 50% reconstitution	1
			50% or less reconstitution	0
**VI**	**Bonding of repair cartilage to denovo subchondral bone:**		complete and uninterrupted	2
			<100% but >50% recon	1
			<50% complete	0
**VII**	**Safranin O staining:**		> 80% homogenous positive stain	2
			40-80% homogenous positive stain	1
			<40% homogenous positive stain	0
			**TOTAL SCORE**	**Max 27**

*Statistical analysis*: Statistical significance between groups for each end point was determined using a one-way analysis of variance (ANOVA) and Bonferroni's post hoc test. Where data sets within groups were not found to be normally distributed, a non-parametric Kruskal-Wallis test was instead used, with a post hoc Dunns multiple comparisons test. A level of *p* < 0.05 was accepted as significant in all analyses. GraphPad Prism 5 statistical software package (Graphpad Software Inc., La Jolla, CA) was used for graph production and data analysis.

## Results

**Figure 1:**
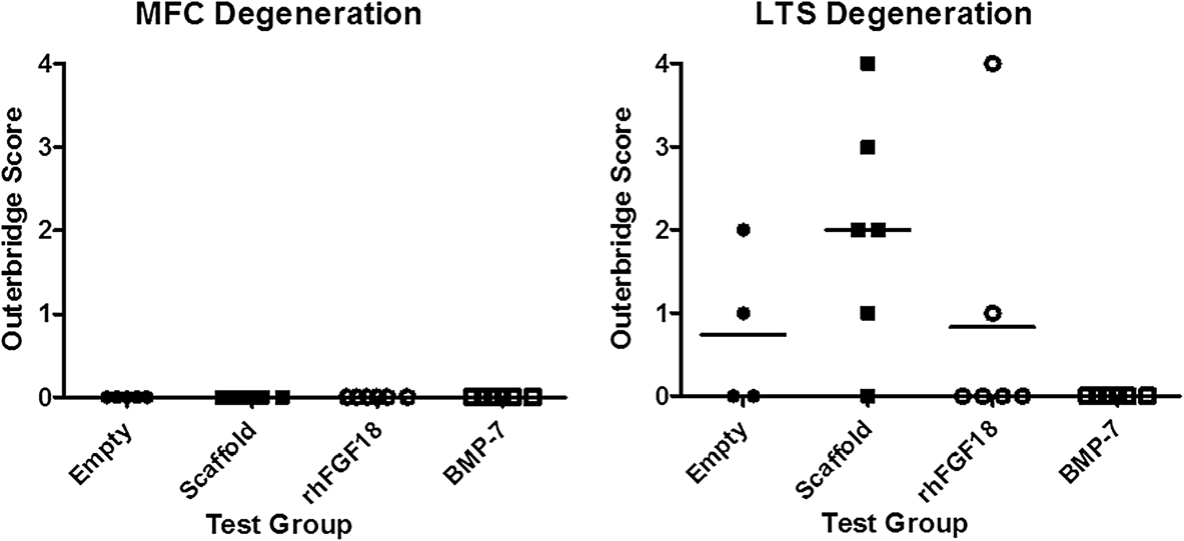
**Macroscopic observation of degenerative change in the MFC and LTS at necropsy.** The horizontal bars represent the mean value. Non-parametric analysis shows a statistically significant difference between the degeneration in the BMP-7 group compared with the scaffold only (p = 0.038).

*Gross morphology (Figure*1*)*: At post-mortem two animals in the empty defect group were shown to have sustained joint damage and were excluded from the study; one with an undisplaced fracture of the lateral trochlea sulcus which was deemed to affect healing, and the second a subluxated patella which would have influenced its weight bearing properties. Degenerative change was seen in many animals in the LTS, particularly in the empty defect and scaffold only groups. Non-parametric analysis showed a statistically significant difference between the degeneration in the BMP-7 group compared with the scaffold only (p = 0.038). Two animals in the BMP-7 group were noted to have osteophyte formation on the MFC.

**Figure 2:**
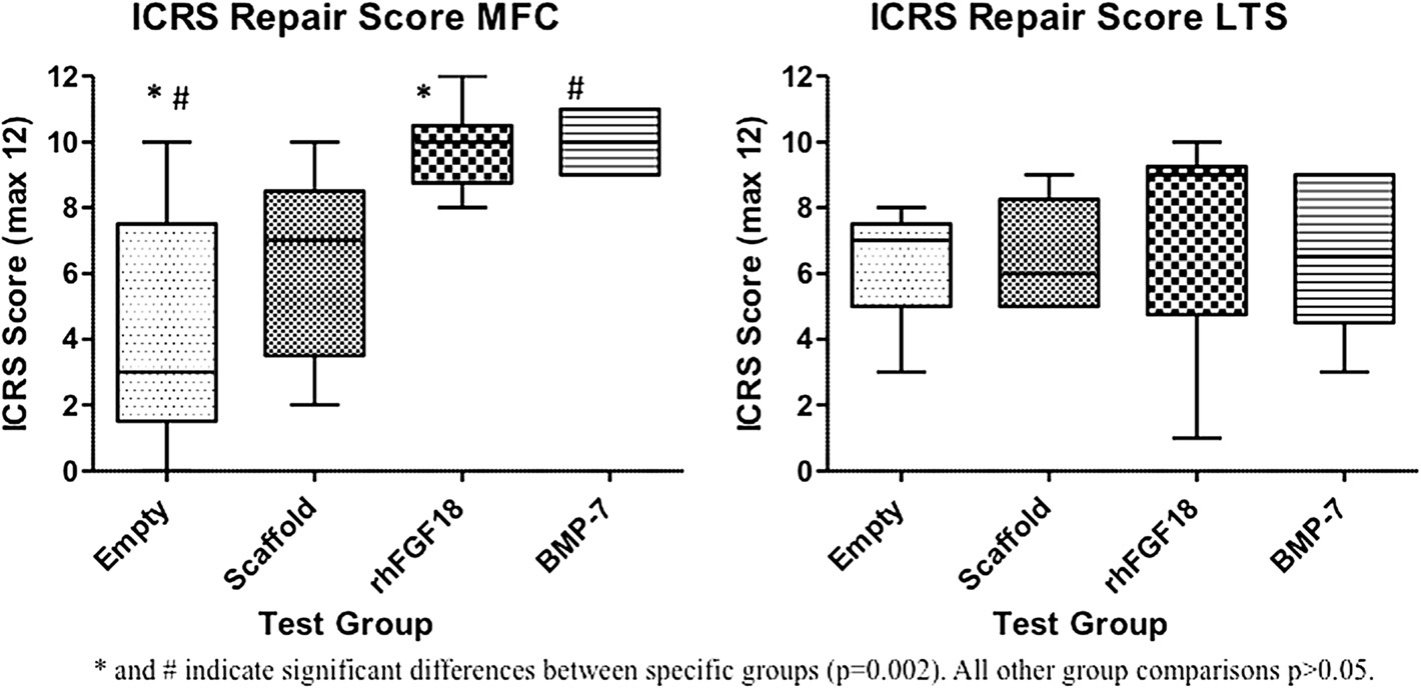
**International Cartilage Repair Society gross repair score of MFC and LTS.** Box and whisker plots represent the maximum and minimum values, the interquartile range with the horizontal bar representing the median value. Statistically significant differences were noted between the empty defects and rhFGF18 and BMP-7 in the MFC (p = 0.002) while no difference was found between groups in the LTS (p > 0.05).

*ICRS repair score (Table*4*and Figure*2*)*: Both rhFGF18 (mean 9.83, 95% CI 8.43-11.23) and BMP-7 (10, 9.06-10.94) had significantly improved ICRS repair scores in the MFC compared to empty defects (4.2, 0–8.80) (p = 0.002). A trend towards improved repair was seen with both growth factors in comparison to the scaffold only but no statistically significant difference existed between groups (p > 0.05). No differences were noted in the LTS.

**Table 4:** **Mean ICRS Repair Score for each category**

	**Empty defect**		**Scaffold only**		**rhFGF18**		**BMP-7**	
	**MFC**	**LTS**	**MFC**	**LTS**	**MFC**	**LTS**	**MFC**	**LTS**
**I. Degree of defect repair**	2.00	2.60	2.83	2.20	3.83	1.60	4.00	6.40
**II. Integration to border zone**	1.20	2.50	2.17	2.67	3.67	1.33	3.50	6.50
**III. Macroscopic appearance**	1.00	3.17	1.33	2.33	2.33	1.83	2.50	7.33
**MEAN TOTAL SCORE**	**4.20**	**2.67**	**6.33**	**2.50**	**9.83**	**1.00**	**10.00**	**6.50**
**p < 0.05**	* #				*		#	

* and # indicate significant differences between specific groups p = 0.002. All other group comparisons p > 0.05.

*Mechanical indentation (Table*5*)*: In the MFC, no differences existed between the treatment groups in the mean percentage stiffness of the contralateral limb (p = 0.31). In the LTS, BMP-7 was significantly less stiff than both rhFGF18 and the empty defect when compared to the cartilage of contralateral limb (p = 0.003). However, the empty defects in the MFC were noted to have higher mean stiffness measurements than normal.

**Table 5:** **Mechanical testing data**

	**Empty defect**		**Scaffold only**		**rhFGF18**		**BMP-7**	
	**MFC**	**LTS**	**MFC**	**LTS**	**MFC**	**LTS**	**MFC**	**LTS**
**Mean**	126.4	85.4	97.2	79.35	99.42	84.1	90.5	64.35
**Std. Deviation**	66.78	13.79	13.8	8.072	7.348	6.958	9.479	7.118
**Std. Error**	29.86	6.167	5.633	3.296	3	2.841	3.87	2.906
**Lower 95% CI**	43.46	68.28	82.72	70.88	91.71	76.8	80.55	56.88
**Upper 95% CI**	209.3	102.5	111.7	87.82	107.1	91.4	100.4	71.82
**p < 0.05**		*				#		* #

* and # indicate significant differences between specific groups p = 0.003. All other group comparisons p > 0.05.

**Figure 3:**
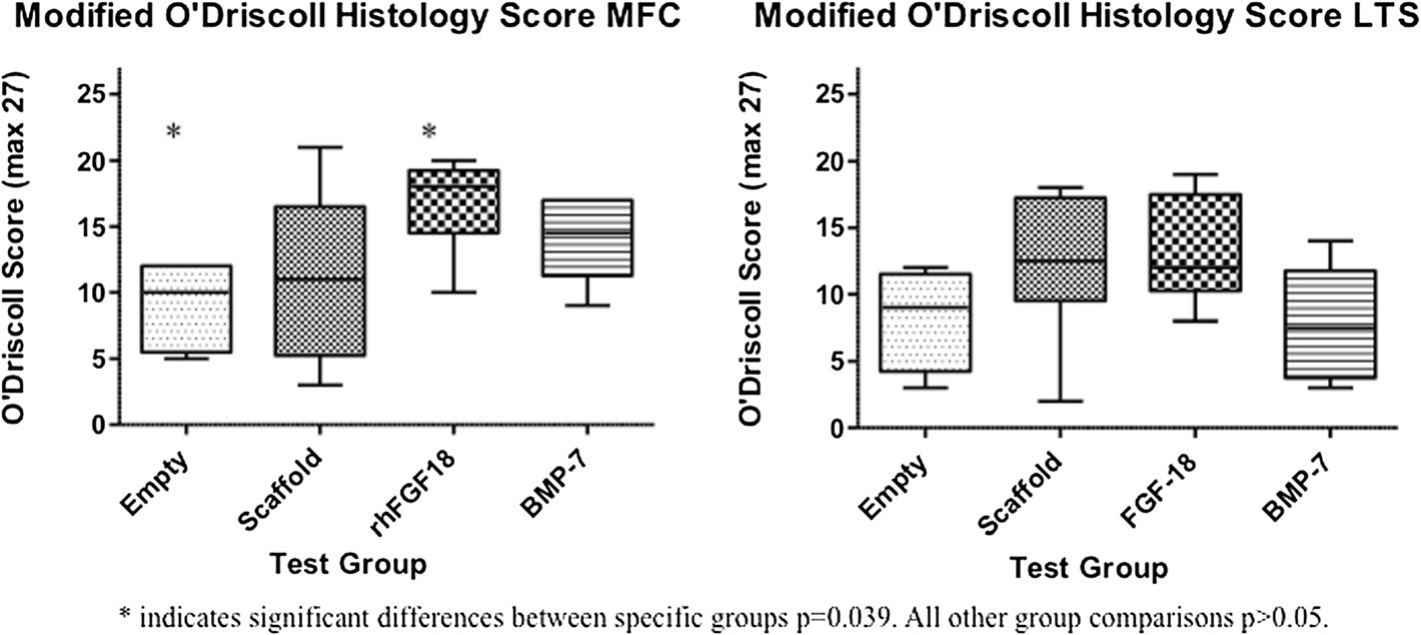
**The modified O’Driscoll score for both MFC and LTS (p > 0.05) showing comparison between groups.** Statistically significant differences were observed between the rhFGF18 and empty defects in the MFC (p = 0.039) with no differences observed in the LTS between groups. Box and whisker plots represent the maximum and minimum values, the interquartile range with the horizontal bar representing the median value.

**Figure 4:**
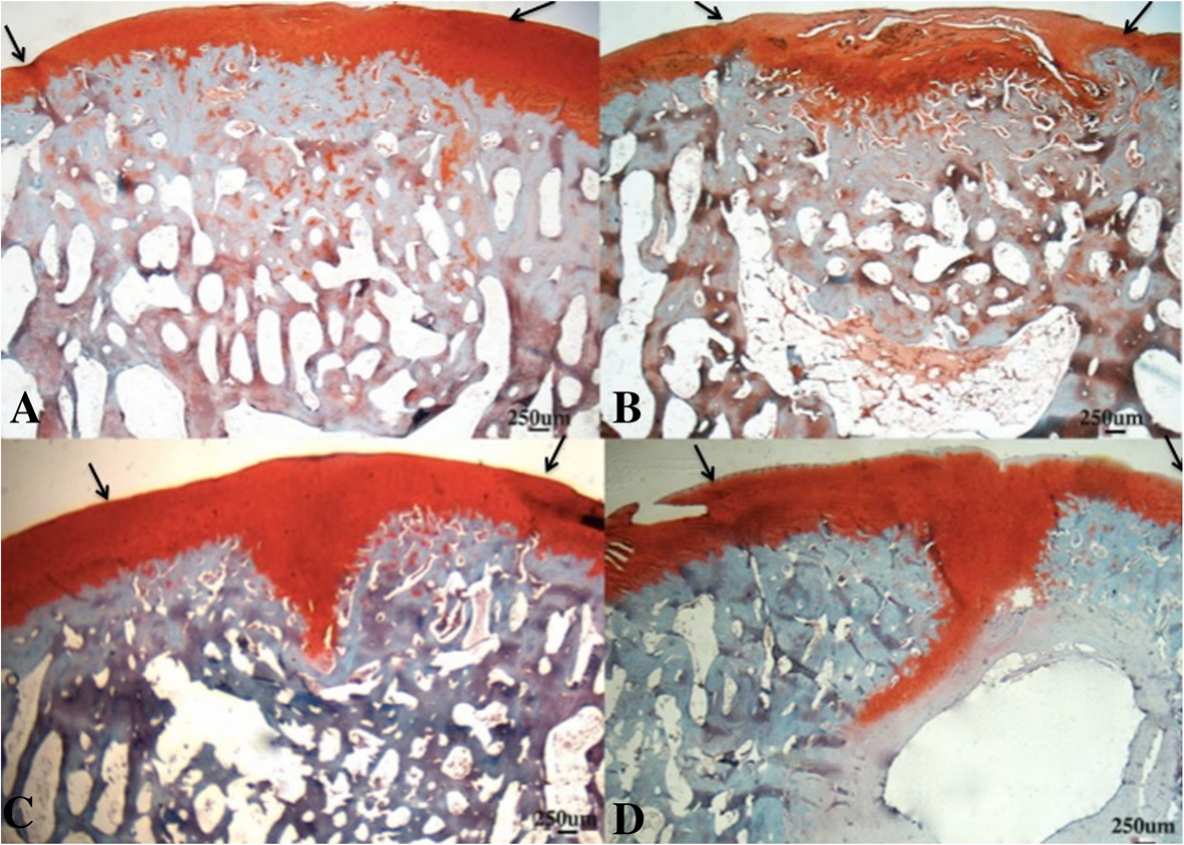
**Histological sections stained with Safranin O. A)** Empty defect, **B)** Scaffold alone, **C)** Scaffold + rhFGF-18, **D)** Scaffold + BMP-7. In A, B and C there is good tissue fill. Differences in subchondral bone formation can be observed. The empty defect A shows subchondral hypertrophy, whilst in C and D there appears to be a cartilage cleft, with significant proteoglycan staining extending down into the subchondral bone. In D there is a large subchondral cyst. The arrows denote the margins of the defect.

**Figure 5:**
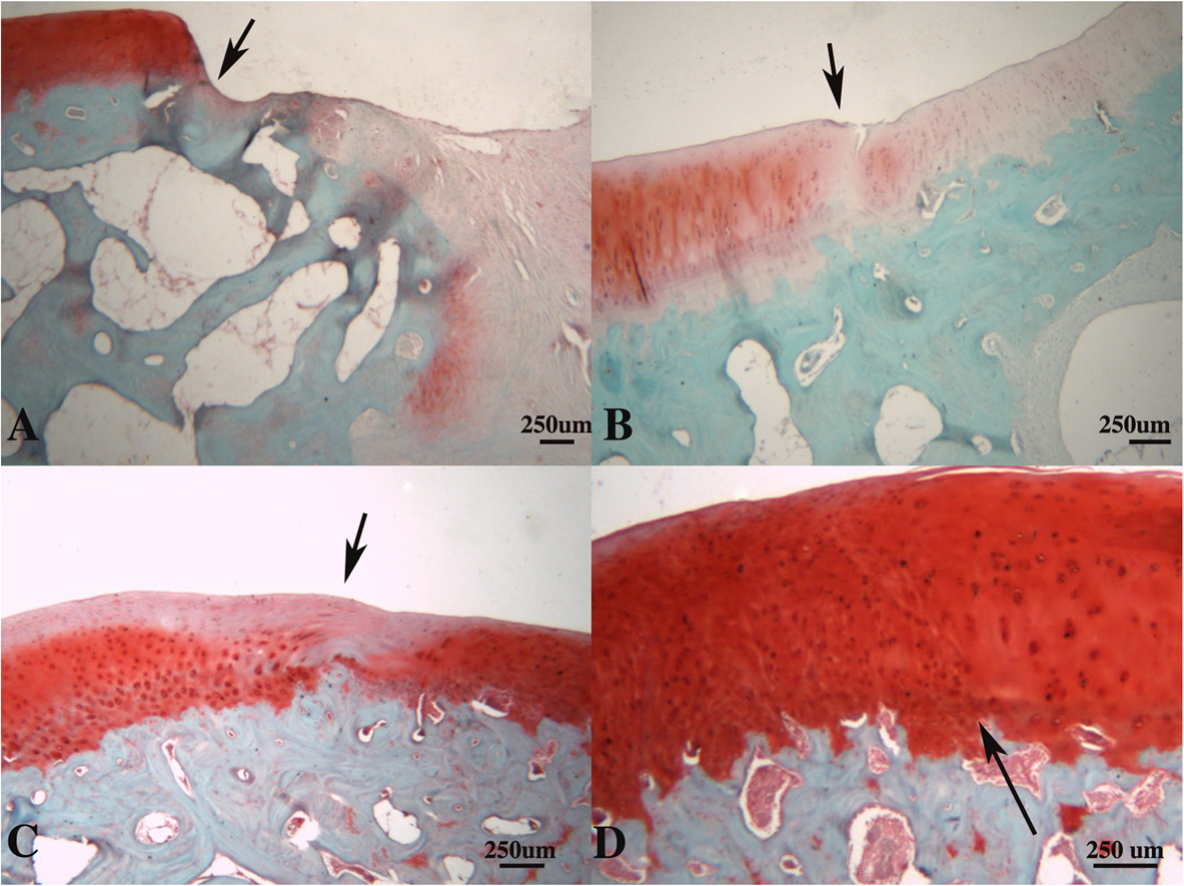
**Representative sections showing different grades of integration of repair tissue to parent cartilage.** Sections stained with Safranin O. Black arrows indicate the junction between repair tissue and parent cartilage. **A)** Poor repair tissue in the defect and no integration between this tissue and the parent cartilage. **B)** There is integration in this section but a hypocellularity at the junction with a small area of fissuring on the articular surface. **C)** Improved integration but hypocellularity at junction. **D)** Excellent integration between repair tissue (left hand side of arrow) and parent tissue (right hand side of arrow).

**Figure 6:**
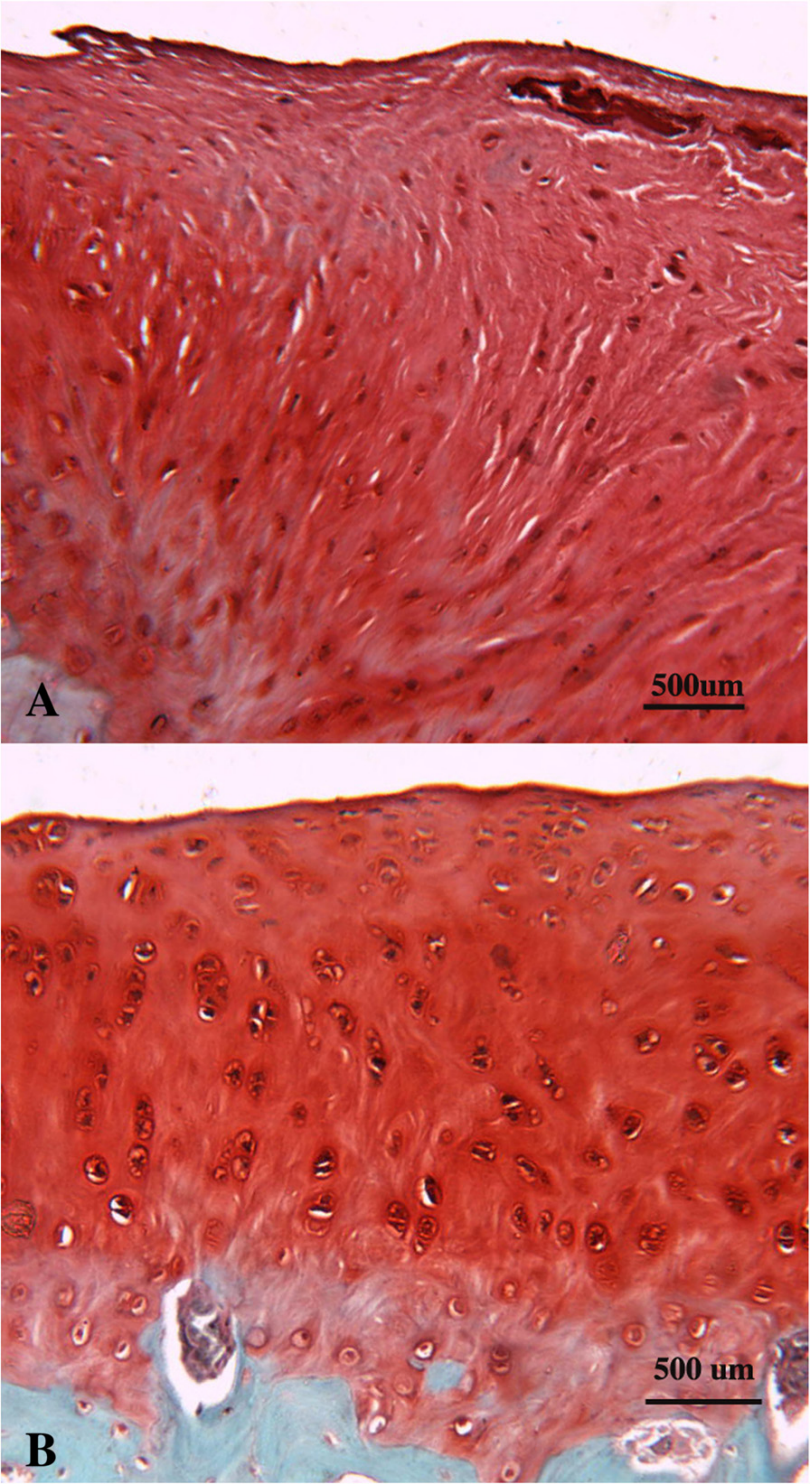
**Histological sections stained with Safranin O. A)** Fibrocartilagenous repair tissue typical of that seen in empty defects, scaffold alone and BMP-7, **B)** Hyaline cartilage-like repair tissue typical of that seen in scaffold + rhFGF-18 treated defects.

**Figure 7:**
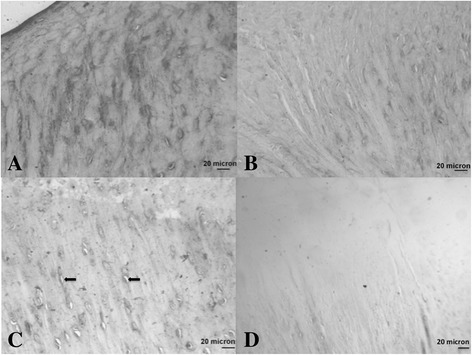
**Histological sections following immunohistochemistry to detect type VI collagen. A)** Empty defect, **B)** Scaffold alone, **C)** Scaffold + rhFGF-18, **D)** Scaffold + BMP-7. In A, where there is a fibrocartilagenous repair, there is little pericellular matrix staining with marked inter-territorial matrix staining. In C there is marked pericellular staining (black rings around the cells) indicating a more hyaline cartilage like repair tissue.

*Histology (Table*6*and Figure*3*):* A significantly higher modified O’Driscoll score was seen in the rhFGF18 group (mean16.83, 95% CI 13.65-20.61) in the MFC when compared to the empty defect (9, 4.88-13.12) (p = 0.039) (Figure 3). A similar trend was observed in the LTS, with both the rhFGF18 and scaffold only groups scoring higher than the empty defect, however no statistical significance was reached. The BMP-7 group showed the worst results of all in the LTS with a wide variability (7.83, 3.46-12.21) **Tissue repair:** Tissue was seen to have filled the entire original defect in the majority of animals (Figure 4). Good tissue fill was noted in both the cartilaginous and osseous parts of the repair in the empty, scaffold only and rhFGF18 treated groups. In the BMP-7 treated group, poor defect fill was seen in four of the six LTS defects with very poor Safranin O staining in the other two defects. In the MFC, the proteoglycan deposition was improved as seen by increased Safranin O staining; however the thickness of the cartilage layer was mostly reduced in comparison to the native cartilage. In addition, two of the six BMP-7 treated MFC defects contained large subchondral cysts (Figure 4D). In the empty defect and scaffold only groups, moderately positive Safranin O staining was seen in MFC defects, with relatively poor staining in the LTS in scaffold only groups. In the rhFGF18 group excellent Safranin O staining was present throughout the chondral portion of all defects indicating good proteoglycan production. Good integration of the repair tissue to the parent cartilage was seen in the scaffold only and rhFGF18 groups, however in the BMP-7 group there was poor lateral integration (Figure 5). **Histology:** In empty defects there was disorganised cartilage tissue within the defect, consistent with a fibrocartilagenous repair (Figure 6A). In the scaffold only and BMP-7 groups there was more organisation of the repair cartilage, albeit in defects with poor tissue fill as noted above. In the lateral margins of the repair tissue a more hyaline cartilage structure was noted, with chondrocytes in lacunae arranged in columns in the deep zone, with more flattened chondrocytes in the superficial zone. A similar histological picture was noted in the rhFGF18 group although the organised hyaline like cartilage (Figure 6B) was present over a larger region of the repair tissue with only a central zone showing fibrocartilagenous characteristics. **Immunohistochemistry:** In all empty defects there was strong type I collagen staining throughout the repair tissue. In the scaffold only and BMP-7 groups both type I and type II collagen staining was detected. In the rhFGF18 group there was negative type I collagen staining and strong type II collagen. No empty or BMP-7 treated defects stained positive for pericellular type VI collagen, pericellular type VI collagen was only noted in the lateral margins of the repair tissue in the scaffold only group while there was strong pericellular type VI collagen throughout the majority of the rhFGF-18 defects (Figure 7).

**Table 6:** **Mean Modified O’Driscoll score for each category**

		**Empty defect**		**Scaffold only**		**rhFGF18**		**BMP-7**	
		**MFC**	**LTS**	**MFC**	**LTS**	**MFC**	**LTS**	**MFC**	**LTS**
**I**	**% Hyaline Cartilage:**	0.80	1.00	1.00	1.67	3.00	2.00	1.33	0.00
**II**	**Structural Characteristics:**								
	**A. Surface Irregularity:**	0.20	0.75	1.17	1.83	1.83	1.33	0.67	0.83
	**B. Structural integrity:**	0.40	0.50	0.67	1.00	1.17	0.50	0.83	0.50
	**C. Thickness:**	0.80	0.75	0.83	0.83	1.33	0.67	1.00	0.33
	**D. Bonding to adjacent cartilage:**	1.20	1.00	2.00	1.50	1.83	1.67	2.00	1.17
**III**	**Freedom from cellular changes of degeneration:**	0.60	0.25	0.67	1.00	0.83	0.83	1.00	0.50
**IV**	**Freedom from degenerate changes in adjacent cartilage:**	1.80	1.75	2.17	1.50	2.33	2.33	2.50	2.33
**V**	**Reconstitution of subchondral bone:**	1.00	0.75	0.33	0.67	0.67	0.83	1.17	0.50
**VI**	**Bonding of repair cartilage to denovo subchondral bone:**	1.60	1.00	1.33	1.67	1.83	1.67	2.00	1.33
**VII**	**Safranin O:**	0.60	0.50	1.00	1.00	2.00	1.33	1.50	0.33
	**MEAN TOTAL SCORE**	**9.00**	**8.25**	**11.17**	**12.70**	**16.83**	**13.20**	**14.00**	**7.83**
	**p < 0.05**	*				*			

* indicates significant differences between specific groups p = 0.039. All other group comparisons p > 0.05.

The histology and immunohistochemistry results indicated that, in the empty group there was a fibrocartilage repair, in the scaffold only and BMP-7 defect groups there was a predominately fibrocartilage repair with some evidence of hyaline cartilage at the lateral margins of the defect. In the rhFGF-18 treated group there was a predominately hyaline cartilage repair with fibrocartilage in the centre of the repair.

## Discussion

This study has shown that the addition of rhFGF18 to a collagen-GAG osteochondral scaffold can produce objective improvements in osteochondral repair tissue in sheep, when compared to defects either left empty, treated with scaffold alone or when the scaffold is combined with BMP-7. The addition of rhFGF18 resulted in statistically significant improvements in gross appearance and histological score over empty defects in the MFC. The combination of scaffold plus rhFGF18 was found to produce similar mechanical properties to native articular cartilage, in contrast to the BMP-7, which was found to be statistically less stiff than both the empty defect and rhFGF18 in the LTS. Histologically, excellent tissue fill of the defects, lateral integration, integrity of neocartilage, and expression of hyaline cartilage markers, such as proteoglycan, type II collagen and pericellular type VI collagen was also observed to a greater degree with rhFGF18. Furthermore, along with BMP-7, rhFGF18 may provide a protective role against perilesional degeneration following osteochondral defect production in the patellofemoral joint and warrants further investigation.

These findings support those of previous studies investigating the role of FGF18 in chondrogenesis. Ellsworth et al. [11] have shown that local delivery of adenovirus expressing FGF18 into the pinnae of nude mice increased the formation of auricular cartilage with high levels of collagen type II and proteoglycan being expressed. Further evidence of its chondrogenic effect were demonstrated by the same group when rhFGF18 was injected intravenously into rats, stimulating expansion of a number of cartilaginous sites including trachea, spine and articular cartilage [18]. In an osteoarthritis small animal model, three injections of rhFGF18 were administered biweekly into rat joints 21 days following meniscus transection [9]. Hyaluronic acid was used both as a carrier vehicle for rhFGF18, and as the control injection. At 6 weeks histological sections representative of the treatment groups showed areas of chondrogenesis with increased proteoglycan deposition and integration of the repair tissue with the native cartilage matrix. Although the repair tissue was not hyaline cartilage, it did show cellular organisation and proteoglycan deposition similar to hyaline cartilage without any evidence of angiogenesis. A repair tissue, originating from the joint margins was also noted, migrating over the cartilage surface. These responses were not seen within the normal rat knee joints (i.e. those which were not subjected to meniscus transection), indicating that rhFGF18 may exert its anabolic effects only on damaged cartilage. A more recent study by Lohmander et al. [19] studied the effects of intra-articular rhFGF18 injected into osteoarthritic joints in a double blind, placebo controlled randomised controlled trial. A statistically significant reduction in joint space narrowing was noted in the lateral tibiofemoral compartment, yet no changes were noted in the medial compartment, nor were changes noted in the patient reported outcome scores. This may indicate that the mechanism of action of rhFGF18 is in some way determined by the mechanical environment, as medial OA is most often associated with varus alignment and relative offloading of the lateral compartment of the knee. The above findings may have particular value in articular cartilage repair, as in the clinical scenario a mixed picture of acute injury on a background of degenerative disease can often be seen. During articular cartilage repair surgery, it is often the case that the perilesional cartilage is diseased, yet the de novo repair tissue is expected to integrate with it forming functional tissue. Furthermore, it is common practice to ‘offload‘ the repair tissue with a realignment osteotomy to improve the mechanical environment. The possibility of a using a growth factor to promote healing and repair within both the defect and perilesional tissue is therefore appealing.

Surprisingly, BMP-7 performed relatively poorly in this experimental study. BMP-7 is considered to be one of the major anabolic growth factors of articular cartilage [20]. Although the gross repair scores were statistically superior to the empty defect, two animals showed osteophyte formation, a factor, which has been noted in a number of other studies incorporating BMPs [21,22]. The histology in the BMP-7 group, particularly the LTS, was very poor. The majority of defects had poor tissue fill which stained poorly for proteoglycan with architecture mostly resembling fibrous tissue. In the MFC, two of the animals were observed to have large cysts present within the subchondral bone, which would likely provide sub-optimal clinical results. As a result of this tissue repair, the mechanical results were significantly inferior to both the rhFGF18 and empty defect groups.

Most of the literature on BMP-7 suggests that it has a acts as an anabolic growth factor, particularly for chondrocyte homeostasis and repair [6]. However, in this model, the main cell type driving the repair process may not be the chondrocyte *per se*, but is perhaps a mesenchymal stem cell population, resident within the subchondral bone or invading blood vessels, which may not be as responsive to BMP-7 treatment. Knippenberg et al. [23] have shown a preference of BMP-7 to direct chondrogenic differentiation of adipose derived MSC’s, compared to an osteogenic lineage by BMP-2. It therefore may not be as potent a growth factor in osteochondral repair as rhFGF18. Alternatively, the dose of BMP-7 used in this model may not have been optimal. The dose of 100 μg per scaffold was chosen based upon personal communication with experts in the field who have previously studied OP-1 (BMP-7) in in-vivo models of cartilage repair [6]. Ideally, further studies should be performed to study different concentrations of growth factor within this model to identify the optimal dose. It should also be noted that the BMP-7 used in this study was not what has been used in other studies provided by Stryker Biotech (Stryker, Kalamazoo, MI), which may have an influence on its biological efficacy.

The positive effects of combining rhFGF18 with a biphasic collagen-GAG scaffold to enhance osteochondral repair is potentially very interesting. rhFGF18 is known to exert an effect on chondrogenesis, as well as osteogenesis [24], so clearly has potential to influence healing across the osteochondral niche. One of the drawbacks of synthetic polymer scaffolds has been reported to be delayed bone formation [25], therefore the addition of a growth factor into the construct, which may speed up the bone regeneration process would be helpful. The ability to control the simultaneous repair of osteochondral tissue is yet to be determined [26]. With increasing time there is a concern that a process of endochondral bone formation is underway, which could result in an advancing tidemark, chondrocyte hypertrophy and calcification of the articular cartilage component of the osteochondral unit. The fact that FGF18 seems to inhibit growth plate chondrocytes may be somewhat protective of that process, however further studies with longer time points until sacrifice would be required to examine this further.

Limitations of this study include that the dosing schedule may not have been optimal, with further injections of rhFGF18 possibly providing greater benefit. The study time also was only 6 months duration. A number of studies have shown that articular cartilage repair can take upwards of one year for maturation [27], therefore having animals out to 12 months to see if these changes observed become more pronounced or equilibrate, would be of interest. The outcome measures used included a surrogate measure of stiffness and semi quantitative histological analysis. Obtaining objective measurements of stiffness and a biochemical analysis of repair tissue would be advantageous, however would have required a greater numbers of animals. As this was a pilot study, the number of animals included was deliberately reduced and therefore this extra analysis was not possible. Furthermore, it is not clear if the findings, which did not show statistical significance, could be secondary to type II error. Further studies are therefore warranted with larger numbers to ascertain the full potential of the drug.

## Conclusion

This study has demonstrated that the combination of rhFGF18 and a collagen-GAG osteochondral scaffold can produce objective improvements in osteochondral repair when incorporated in an acute osteochondral defect model in sheep, although topographic variation may exist between the MFC and LTS. The use of rhFGF18 as a target molecule for articular cartilage repair and osteoarthritis treatment warrants further investigation.
